# Paracricetodontinae (Mammalia, Rodentia) from the late Eocene and early Oligocene of south-east Serbia

**DOI:** 10.1007/s12549-017-0317-9

**Published:** 2018-02-24

**Authors:** Andrew A. van de Weerd, Hans de Bruijn, Zoran Marković, Wilma Wessels

**Affiliations:** 1 Department of Earth Sciences, Utrecht University, Princetonlaan 8A, 3584 CB Utrecht, the Netherlands; 2 Natural History Museum in Belgrade, Njegoševa 51, Belgrade, 11000 Serbia

**Keywords:** Rodentia, Paracricetodontinae, Late Eocene, Early Oligocene, South-east Serbia

## Abstract

Three *Paracricetodon* species from the late Eocene locality Buštranje and the Early Oligocene localities Valniš, Strelac-1, -2, -3 and Raljin (south-east Serbia) are described; *Paracricetodon dehmi* Hrubesch, 1957 and two new species: *Paracricetodon stojanovici* and *P. gracilis.* A review of *Paracricetodon* species suggests that the species *P. spectabilis*, *P. cadurcensis*, *P. dehmi*, *P. walgeri* and *P. wentgesi* are primarily distinct in size. *Paracricetodon kavakderensis* and *P. kodjayarmensis* from Turkish Trace are considered junior synonyms of *P. dehmi*. The diversity and abundance of the Paracricetodontinae in the rodent assemblages from Serbia is not known from elsewhere and suggest that they underwent a radiation on the Serbian-Macedonian land area.

## Introduction

This study on the Paracricetodontinae is part of a series dealing with late Eocene and early Oligocene rodent faunas from seven localities in two basins in south-east Serbia. The first paper considers the geological setting, the description of the localities were the fossils have been collected, the methods and the general composition of the faunas (Bruijn et al. in press). This paper was followed by systematic descriptions of the rodent subfamilies of the Diatomyinae (Marković et al. in press) and the Melissiodontinae (Wessels et al. in press). Table 1 shows the rodent taxa present in the faunas from south-east Serbia; *Paracricetodon* occurs in six of the seven Serbian sites. In the late Eocene locality of Buštranje it is present with about 10% of the specimens and in the early Oligocene sites with 30 to 70% of the specimens.

**Table 1:** The composition of the rodent faunas in south-east Serbia

Family	Subfamily	Genus and species	Eocene		Early Oligocene					
			Zvonce	Buštranje	Strelac-1	Strelac-2	Strelac-3	Valniš	Raljin	Total M1- M2
Diatomyidae	Diatomyinae	*Inopinatia balkanica*			7	4	3	49	2	65
Dipodidae	primordial Zapodidae	*Heosminthus borrae*				X	22	20	1	43
Muridae	Pseudocricetodontinae	*Heterocricetodon* nov. sp. A			14	5	6	49	4	78
		*Pseudocricetodon* nov. sp. (small)		29			14			43
		*Pseudocricetodon montalbanensis*			4		23	28	8	63
	Paracricetodontinae	*Paracricetodon dehmi*			3		X	10		13
		*Paracricetodon gracilis* nov. sp.					2	11	?	13
		*Paracricetodon stojanovici* nov. sp.		75	45	26	30	127	9	312
	Pappacricetodontinae	*Witenia* sp.			5		X	2		7
		nov. gen.3 nov. sp. A		601						601
		*Witenia* nov. sp. A		21						21
	Melissiodontinae	cf. *Edirnella* sp. 2			6	1				7
		*Mogilia lautus*			X			34	1	35
		*Mogilia miloshi*	28	30						58
		cf. *Edirnella* sp. 1		4		1				5
		cf. *Edirnella* sp. indet.	X							1
	?Spalacinae	nov. gen.1 sp. A	3							3
Total number of upper and lower M1 and M2 in each locality			31	760	84	37	100	330	25	1368

European as well as Turkish small mammal faunas contain only a single species of *Paracricetodon* Schaub, 1925 (with a possible exception in Heimersheim, Germany; Bahlo 1975). The presence of three *Paracricetodon* species, co-occurring in two of our Serbian assemblages, leads us to review the species included in *Paracricetodon*.

Schaub (1925), in his classical work on Tertiary and extant hamster-like rodents, showed that the species *Cricetodon spectabilis* Schlosser, 1884 and *Cricetodon cadurcensis* Schlosser, 1884 are very similar in dental characteristics and in the shape of their mandibles, but differ essentially in these respects from *Cricetodon* proper and thus merit generic distinction. Consequently, he defined the genus *Paracricetodon*, designated *Cricetodon spectabilis* as the type, allocated the smaller species *cadurcensis* to his new genus and named a third species on the basis of a single mandible of intermediate size, *Paracricetodon confluens.*

Unfortunately, the type specimens of all these three species originate from the karst-fissure fills of the Quercy (France) that were mined at the time, ground up and used as a phosphate fertiliser. The locality data with these specimens in old collections from the Quercy refer to the villages where the mills were located (i.e. Caylux, Moulliac) and give no information on the sites where the fossils came from.

Renewed collecting in the Quercy since 1963, originally by M. Freudenthal and P. Y. Sondaar (at the time Utrecht University and assisted in the field by the first author when he was a junior student) and somewhat later by L. Thaler and collaborators (Université des Sciences et Techniques du Languedoc, Montpellier), has shown that these karst fissures contain faunas that range in age from MP16 to MP28 (Remy et al. 1987). The oldest record of *Paracricetodon* in the Quercy comes from Lebratières 14, an assemblage that has been correlated with MP24 by Remy et al. (1987).

*Paracricetodon* material from the Quercy that has been studied in detail by Vianey-Liaud et al. (1995) comes from the MP 25 localities Garouillas and Rigal Jouet-1. They demonstrate that the only specimen that is still available of the type material of *Paracricetodon cadurcensis* is within the range of variation of the *Paracricetodon* specimens of these two MP 25 localities and provide an emended diagnosis of *P. cadurcensis*. In the meantime, it remains unknown to which Quercy fauna *P. spectabilis* and *P. confluens* belong.

In spite of extensive collecting in stratified as well as in karst deposits of Oligocene age since Schaub’s days, *Paracricetodon* has remained a rare animal in central and western European assemblages. Samples that are large enough to allow the study of the intraspecific variation are known from the early Oligocene of Heimersheim (MP24; Bahlo 1975), Bernloch (MP24?; Hrubesch 1957), St Martin de Castillon (MP24; Hugueney and Adrover 1989–1990), Turkish Thrace (~MP24-25; Ünay-Bayraktar 1989), the Eocene/Oligocene boundary interval of the Lesser Caucasus (de Bruijn et al. 2003) and in south-east Serbia (this paper). The type species *Paracricetodon spectabilis* from the Quercy is, because of its larger size, a probably late member of this genus and from the periphery of its geographical range*.*

Mein and Freudenthal (1971), noting the unique dental characters of *Paracricetodon*, defined the monogeneric subfamily Paracricetodontinae in their classification of the Tertiary Cricetidae of Europe. Since then the content of this subfamily in terms of genera and its phylogenetical position within the Muridae has been disputed, so our usage needs explaining.

Ünay-Bayraktar (1989) defined the genus *Trakymys* and included that in the Paracricetodontinae. In the same publication, she suggested that the Paracricetodontinae and the Melissiodontinae Schaub, 1925 (with the genera *Melissiodon* Schaub, 1920 and *Edirnella* Ünay-Bayraktar, 1989) are closely related and therefore are members of the same family. Freudenthal et al. (1992) did not agree with this arrangement, maintained the Melissiodontidae as a monogeneric family and transferred the genus *Edirnella* to the Paracricetodontinae. McKenna and Bell (1997) classified all the European Oligocene cricetids, except *Melissiodon*, as well as a number of North American genera in the Paracricetodontinae. They also followed Freudenthal et al. (1992) by including *Edirnella* in the Paracricetodontinae. Finally Kalthoff (2006) discussed the classification of the Eurasian Oligocene Muridae on the basis of the microstructure of the enamel of the lower incisors. The incisor enamel of *Melissiodon* (her type 1) appeared to be the most primitive known in murids and very different from the derived four-layered (type 8) that characterises the paracricetodontines *Paracricetodon* and *Trakymys* (Martin 1997; Kalthoff 2000). Unfortunately the isolated incisor studied by Kalthoff, that was supposed to belong to *Edirnella*, appeared to be a misidentified *Trakymys* tooth (Wessels et al. in press).

Our usage of the subfamily Paracricetodontinae is restricted to bunodont cricetids in which the lingual sinus of the M1 and M2 is directed posteriorly and the microstructure of the lower incisor shows four layers, and includes the genera *Paracricetodon* Schaub, 1925 and *Trakymys* Ünay-Bayraktar, 1989. The melissiodontines (including *Melissiodon* and *Edirnella*) from the late Eocene - early Oligocene rodent assemblages from Serbia are much more diverse than hitherto assumed and represent a clade that is not closely related to the Paracricetodontinae (Wessels et al. in press).

The eight formally named *Paracricetodon* species are as follows: *P. spectabilis* (Schlosser, 1884), *P. cadurcensis* (Schlosser, 1884) and *P. confluens* Schaub, 1925 from the Quercy (France), *P. dehmi* Hrubesch, 1957 and *P. walgeri* Bahlo, 1975 from Germany, *P. kodjayarmensis* Ünay-Bayraktar, 1989 and *P. kavakderensis* Ünay-Bayraktar, 1989 from Turkish Thrace and *P. wentgesi* de Bruijn et al., 2003 from the lesser Caucasus (Turkey).

**Fig. 1:**
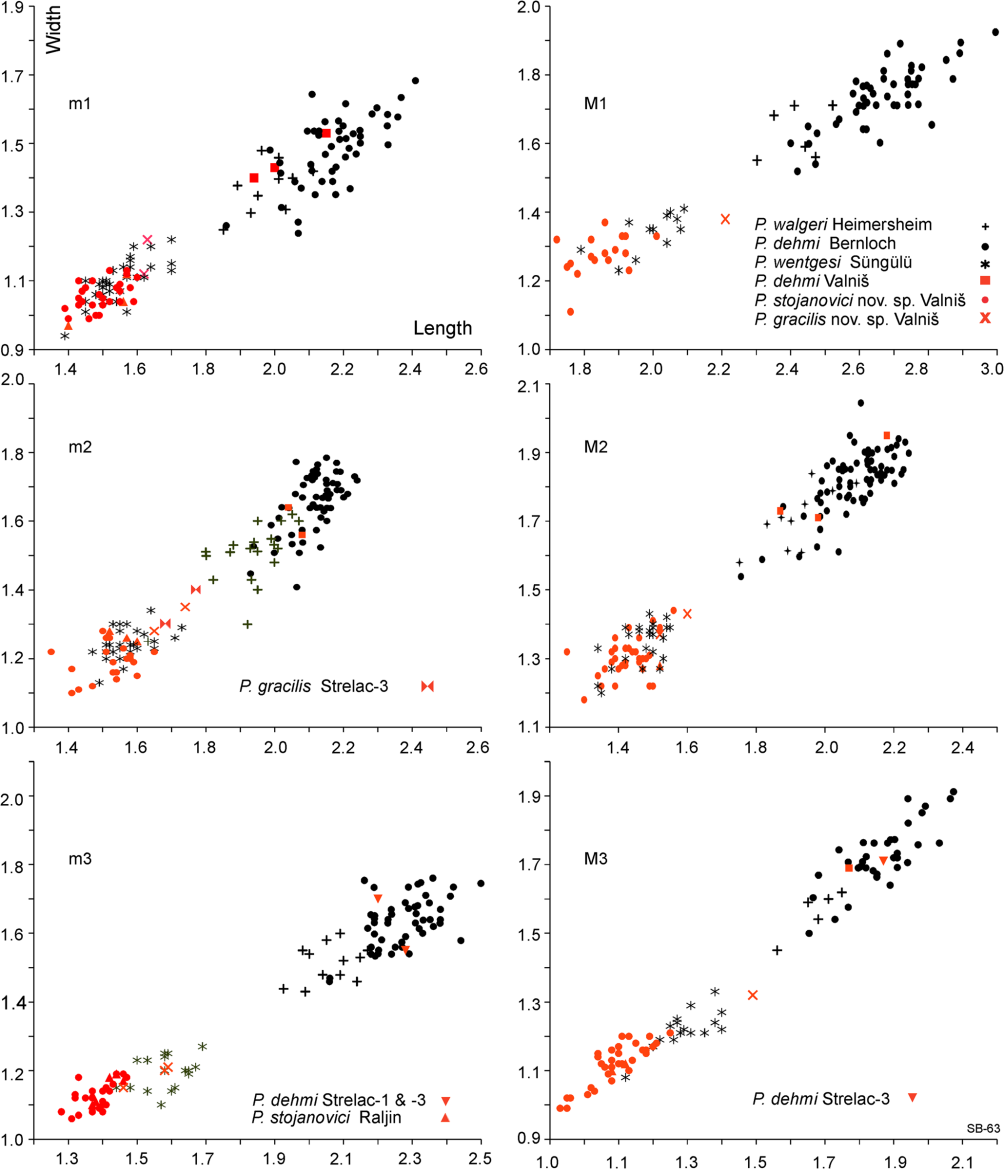
Length-width scatter diagrams of the molars of *Paracricetodon* from south-east Serbia (in red) and from some selected species from other locations (in black)

**Fig. 2:**
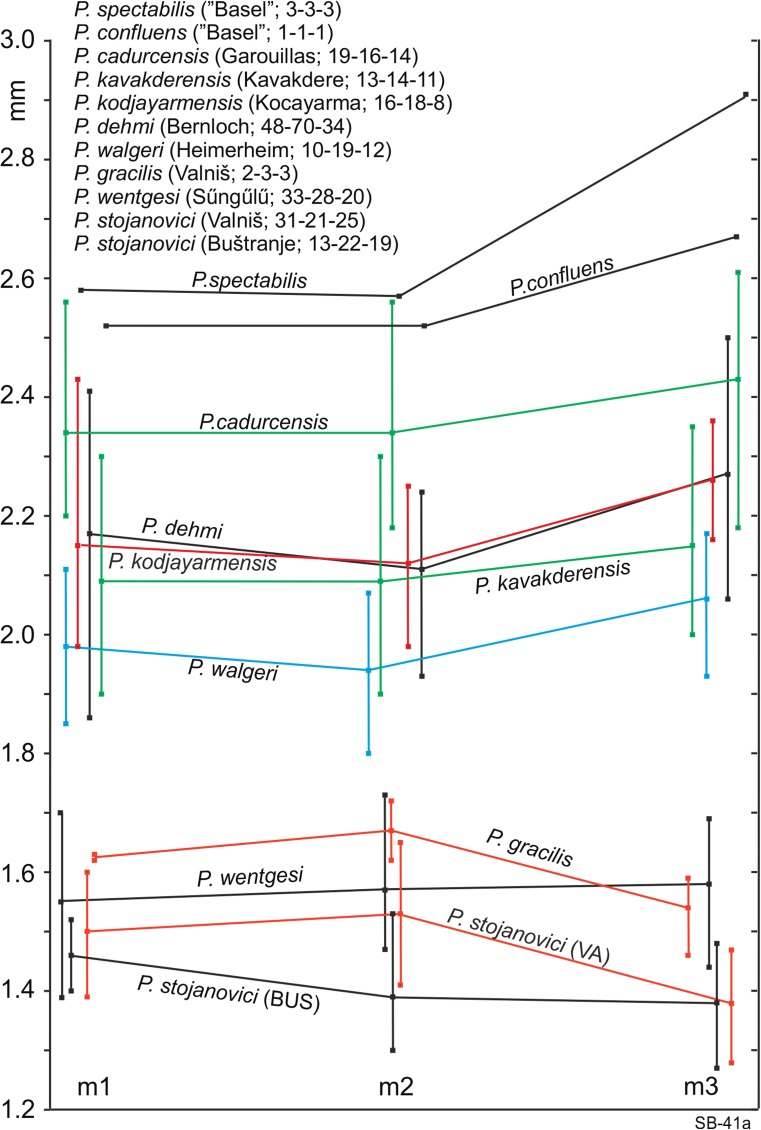
The length of the lower molars in *Paracricetodon* species from different localities. Shown are the average length and the range of the m1, m2 and m3. The numbers between brackets behind the location names represent the numbers of m1, m2 and m3 on which the averages and ranges are based

**Fig. 3:**
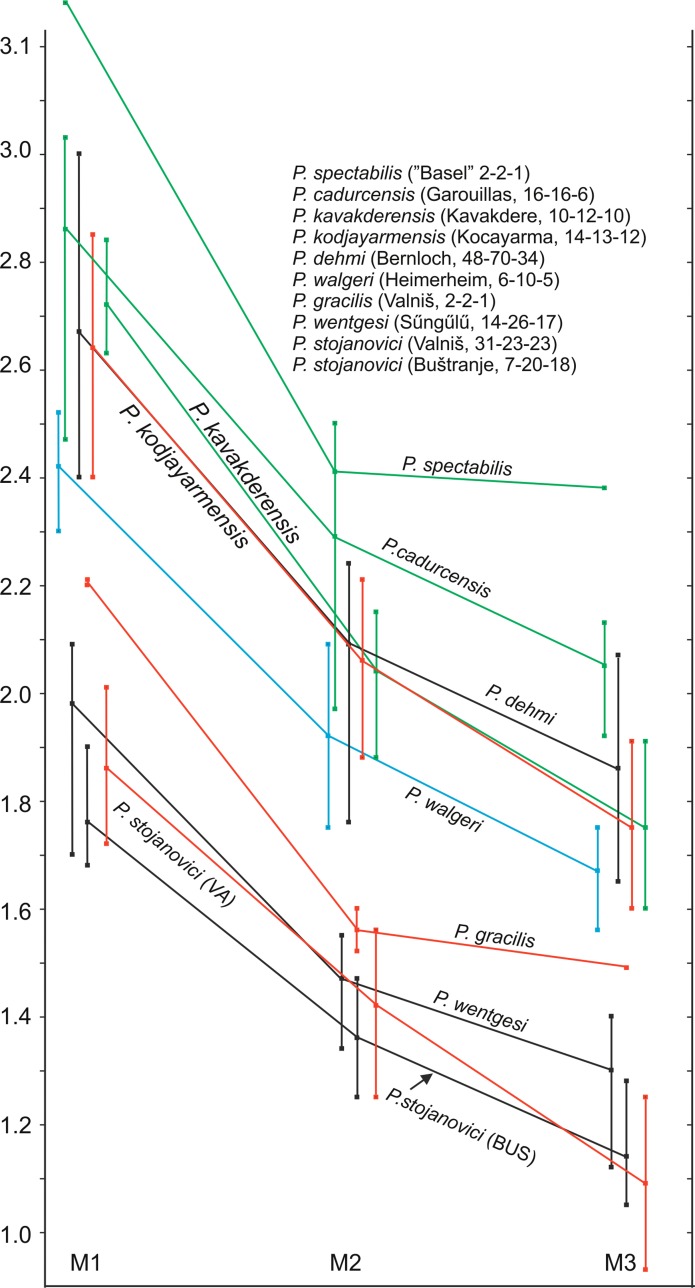
The length of the upper molars in *Paracricetodon* species from different localities. Shown are the average length and the range of the M1, M2 and M3. The numbers between brackets behind the location names represent the numbers of M1, M2 and M3 on which the averages and ranges are based

Figures 1, 2 and 3 clearly show that there are two size groups of *Paracricetodon* species: the small species are known from the Balkan and the lesser Caucasus region only; with the possible exception of the single m2 from Heimersheim in Germany. Figures 2 and 3 give the mean values of the length as well as the observed ranges of the lower respectively upper cheek teeth of the type material and of material of all known species of *Paracricetodon.*

The type material of *Paracricetodon spectabilis*, *P. cadurcensis* and *P. confluens* from the Quercy is very limited, so the intraspecific variation in size and morphology is not known (Figs. 2 and 3).The first well-documented species of *Paracricetodon* found is *P. dehmi* from Bernloch (Hrubesch 1957). From the description of that material we know that the variation in size as well as in morphology within a single association is large. The length-width scatters of Hrubesch have been digitised and shown here in Fig. 1. Hrubesch (1957) states that the teeth from Bernloch, although on average smaller than the three species known at the time, may show dental details that were, until then, used to distinguish these species.

Vianey-Liaud et al. (1995) in their study of the *Paracricetodon* material from Garouillas and Rigal-Jouet-1 confirmed this conclusion and observed that, although the morphological variation within single assemblages is great, the dental morphology remains essentially unchanged in different species; Hugueney and Adrover (1989-1990) came to a similar conclusion. The consequence of these observations is that the species *Paracricetodon spectabilis*, *P. confluens* and *P. cadurcensis*, which are each based on one or very few specimens, can be recognised by size only. Since the lengths of the molar of the type and the only specimen of *P. confluens* are within the expected, but insufficiently known, ranges of the molars of *P. spectabilis* and *P. cadurcensis*, we tentatively consider the name *Paracricetodon confluens* a nomen dubium (Fig. 2). *Paracricetodon* molars that match the size of the type specimen of *P. cadurcensis* are described by Vianey-Liaud et al. (1995), so this species is now adequately known on the basis of the material from the localities Garouillas and Rigal-Jouet-1. *P. cadurcensis* is larger than *P. dehmi* but the size ranges show considerable overlap*.* Specimens that fit the *P. cadurcensis* size range are known from Sineu (Balearic Islands, Spain) and Terrenoire (France, Hugueney and Adrover, 1989-1990).

Figures 2 and 3 clearly show that *Paracricetodon spectabilis*, the type species of *Paracricetodon*, is not only the largest of all the *Paracricetodon* species, but also has the longest M3 relative to the M2, while *P. wentgesi* is the smallest and has, like *P. gracilis*, a short M3 relative to the M2. The relative dimensions of the upper and lower cheek teeth are stable throughout all the species of the genus otherwise. The length and dental morphology of the cheek teeth of *P. dehmi*, *P. kodjayarmensis* and *P. kavakderensis* overlap almost completely. In spite of the minor differences observed by Ünay-Bayraktar (1989), the species from Turkey are therefore tentatively considered to be junior synonyms of *P. dehmi*.

The assemblage from Belgarite 4A (Quercy) in the collection of the Faculty of Earth Sciences of Utrecht University (Fig. 9) fits the type material of *Paracricetodon dehmi* and is considered to represent that species. The lengths of the cheek teeth of *P. dehmi* s.l. show some overlap with those of the larger *P. cadurcensis* and the smaller *P. walgeri*, but the mean values of their cheek teeth differ sufficiently to maintain *P. cadurcensis, P. dehmi* and *P. walgeri* (Figs. 2 and 3). This conclusion is further supported by the total range of the size of these three species, which far exceeds the theoretically expected range within one species. In spite of the reduction of the number of *Paracricetodon* species the overlap in size and morphology between them makes the identification of small samples to the species level difficult if not impossible.

The isolated *Paracricetodon* teeth from the Serbian Pčinja and Babušnica-Koritnica basins represent three species. One of these is small and is common in the Eocene site Buštranje as well as in all five early Oligocene localities in the Strelac area. The second species is represented by a few specimens from the two larger Oligocene assemblages: Valniš and Strelac-1 and has been identified as *Paracricetodon dehmi* Hrubesch, 1957. The third species, also rare, is of medium size; it is present in Valniš and Strelac-3. A single aberrant m3 from Strelac-1 has been tentatively allocated to *P. dehmi*, however, it may document *Trakymys.* In two localities, Strelac-3 and Valniš all three *Paracricetodon* species occur together. As far as we know, western and central European faunas contain only a single species of *Paracricetodon.* A possible exception is Heimersheim (Germany) where next to *P. walgeri* a smaller species is perhaps documented by a small m2 in the scatter diagram (Bahlo 1975: fig. 16). It has a length and width of 1.63 mm and 1.25 mm respectively and is included diagram of Fig. 1, where it is hidden in the point cloud of *P. wentgesi.*

## Material and methods

The isolated cheek teeth of the Paracricetodontinae from south-eastern Serbia are housed in the Natural History Museum in Belgrade (Serbia). A representative set of casts is kept in the collection of the department of Earth Sciences of Utrecht University, the Netherlands.

**Fig. 4:**
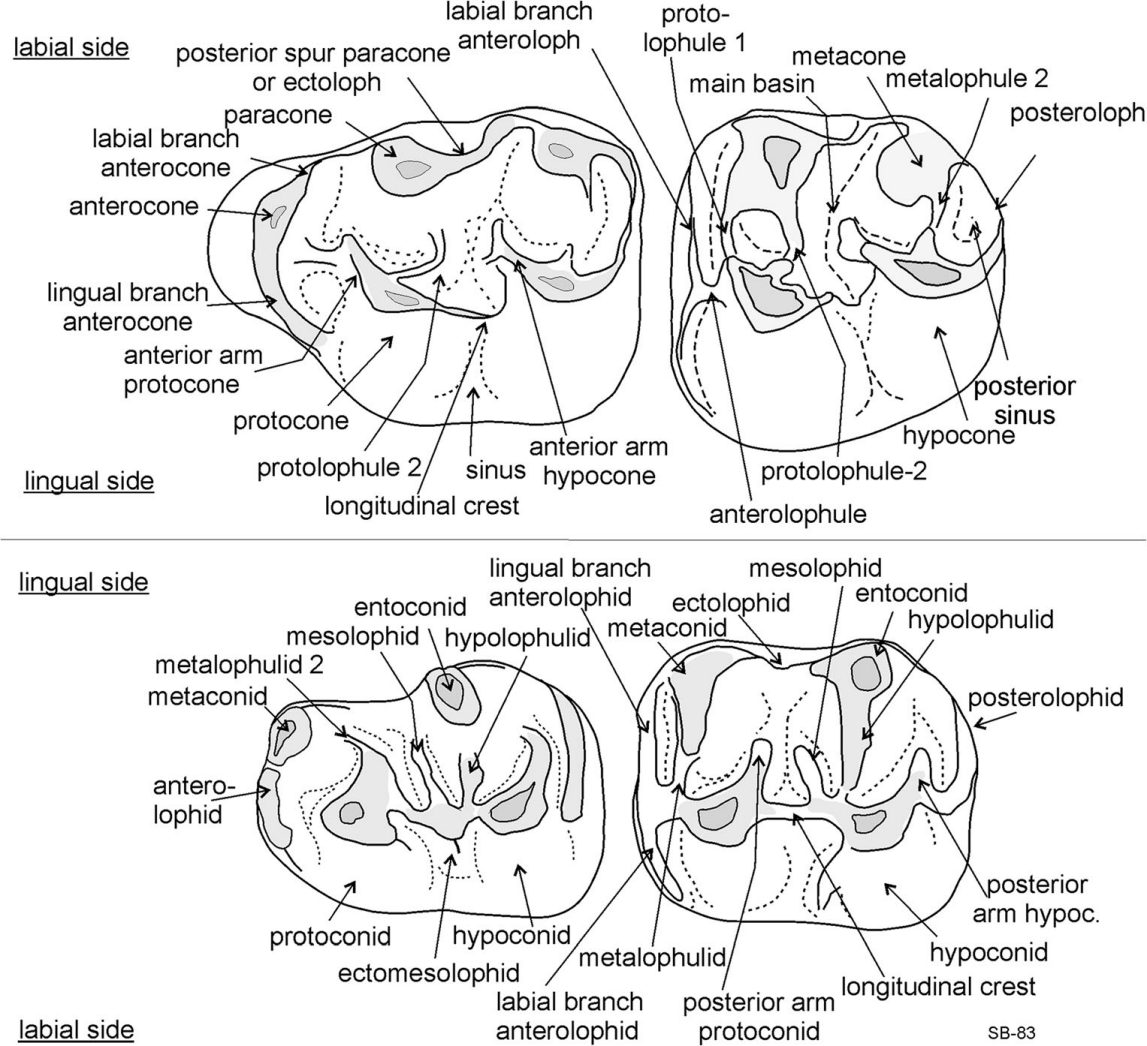
Terminology used in the description of cheek teeth of *Paracricetodon*

The terminology used for the description of the cheek teeth is illustrated in Fig. 4.

The measurements of the teeth have been taken with a Leitz Ortholux measuring microscope with mechanical stage and measuring clocks. The measurements used in the Figs. 2 and 3 have partly been taken from the literature, partly taken recently by colleagues or by our own, so there is a danger that the result is biased by the use of different measuring techniques and/or the usage of different measuring devices. This may in particular be the case for the values of the teeth from Bernloch, because the ranges of the length of the teeth given by Hrubesch (1957) for that sample are larger than expected and it is clear that the sophisticated measuring devices used nowadays were not available in 1957.

The pictures were made using a table-top SEM and a high-resolution SEM. All specimens are figured as left ones. If the original is from the right side, this is indicated by underlining its number on the figure. Lower case letters refer to the lower dentition, upper case letters refer to the upper dentition. Abbreviations for measurements and descriptions are as follows: dext–dextral, L–length, N–number of specimens, R–range of measurements, sin–sinistral and W–width.

The abbreviations used for of the localities are as follows: Buštranje (BUS), Strelac-1 (STR-1), Strelac-2 (STR-2), Strelac-3 (STR-3), Valniš (VA) and Raljin (RA).The codes of the localities that yielded this material are: 031 for Buštranje, 024 for Strelac-1, 025 for Strelac-2, 026 for Strelac-3, 027 for Valniš and 028 for Raljin.

Abbreviations and terminology used in the description of the microstructure of enamel are as follows: HSB–Hunter Schreger band, IPM–inter prismatic matrix, PE–portio externa and PI–portio interna.

Used for comparison are casts of the specimens of *P. spectabilis* (Schlosser, 1884) and *P. cadurcensis* (Schlosser, 1884) that are figured in Schaub (1925), a cast of the type of *P. confluens* Schaub, 1925 and casts of *P. dehmi* Hrubesch, 1957, *P. kavakderensis* Ünay-Bayraktar, 1989, *P. kodjayarmensis* Ünay-Bayraktar, 1989, *P. wentgesi* de Bruijn et al., 2003, *Trakymys saratji* Ünay-Bayraktar, 1989 as well as a sample of isolated *Paracricetodon dehmi* teeth from the Quercy collection made by Freudenthal and Sondaar in 1965–1967 labelled Belgarite 4A (all stored in the collection of Utrecht University, Department of Earth Sciences). Unfortunately the locality names used by the Dutch team for the Quercy fissures that yielded this material have not been harmonised with those used by French teams. This means that their biostratigraphical position within the Quercy sequence worked out by the French teams is not exactly known.

## Taxonomy

Family Muridae Illiger, 1811

Subfamily Paracricetodontinae Mein and Freudenthal, 1971

**Genera included:** *Paracricetodon* Schaub,^1925^ and *Trakymys* Ünay-Bayraktar, 1989

Genus *Paracricetodon* Schaub, 1925

**Type species:** *Cricetodon spectabilis* Schlosser, 1884

**Other species recognised:** *P. cadurcensis* (Schlosser, 1884), *P. dehmi* Hrubesch, 1957 (= *P. kavakderensis* Ünay-Bayraktar, 1989; = *P. kodjayarmensis* Ünay-Bayraktar, 1989), *P. walgeri* Bahlo, 1975, *P. wentgesi* de Bruijn et al., 2003, *P. stojanovici* nov. sp. and *P. gracilis* nov. sp. *Paracricetodon confluens* Schaub, 1925 is considered to be a “nomen dubium” because it is based on a single mandible with m1 – m3 that is intermediate in size between those of *P. spectabilis* and *P. cadurcensis* and lacks species-characteristic dental features.

**Fig. 5:**
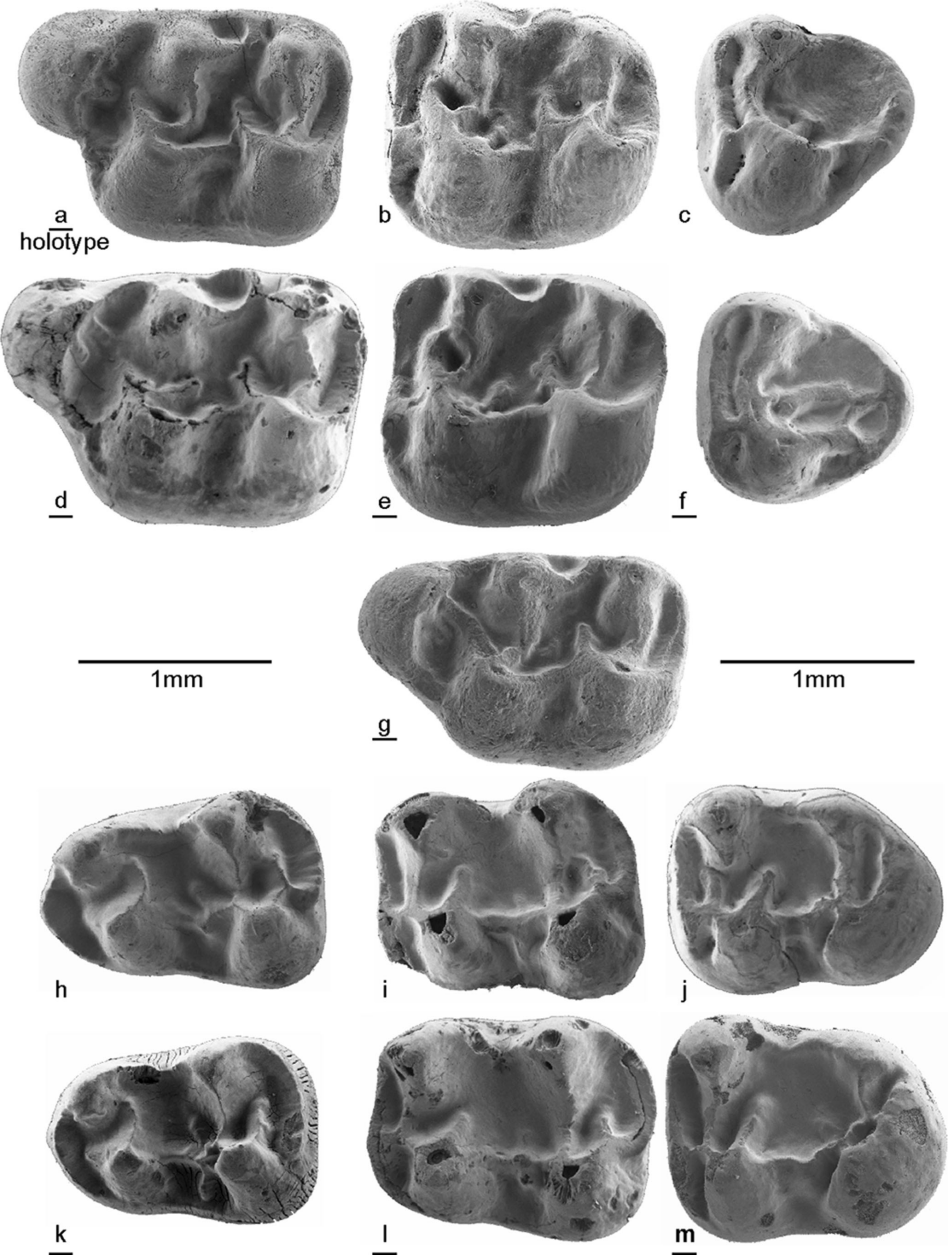
*Paracricetodon stojanovici* nov. sp. from the type locality Buštranje**. a** M1 dext BUS-668 (holotype). **b** M2 sin BUS-682. **c** M3 sin BUS-704. **d** M1 dext BUS-671. **e** M2 dext BUS-694. **f** M3 dext BUS-717. **g** M1 dext BUS-666. **h** m1 sin BUS-722. **i** m2 sin BUS-741. **j** m3 sin BUS-762. **k** m1 dext BUS-732. **l** m2 dext BUS-754. **m** m3 dext BUS-775

*Paracricetodon stojanovici* nov. sp. (Fig. 5, 7a–i, 8a–j,10)

**Derivatio nominis:** The species is named after Jovan Stojanović, in recognition of the way he cared for us during our stays in Babušnica.

**Type locality:** Buštranje.

**Holotype:** an isolated M1 dext.; VA-668 (Fig. 5a).

**Occurrences:** Valniš, Strelac-1, Strelac-2, Strelac-3, Raljin.

**Fig. 6:**
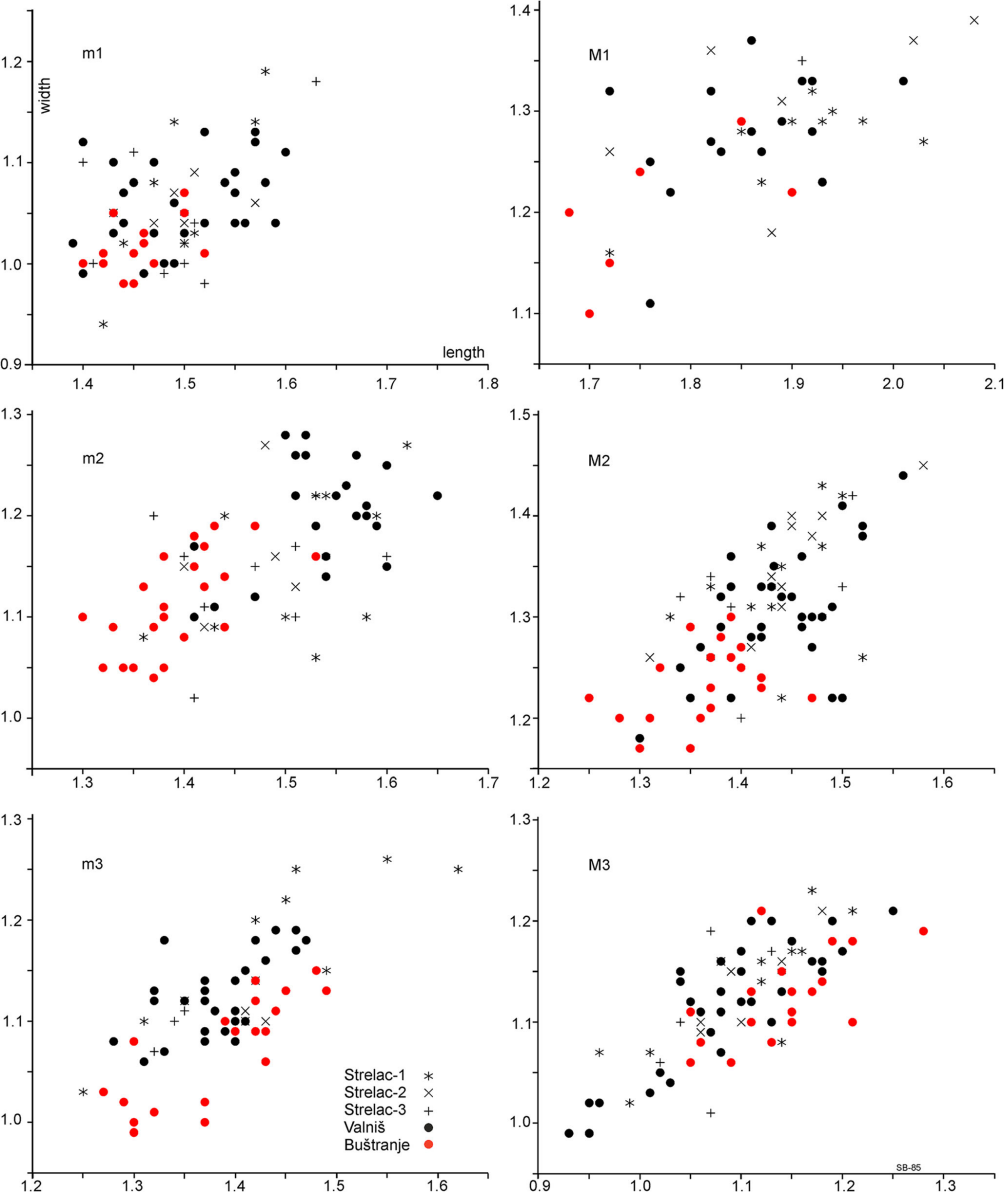
Length-width scatter diagrams of the molars of *Paracricetodon stojanovici* from five localities in south-east Serbia

**Fig. 7:**
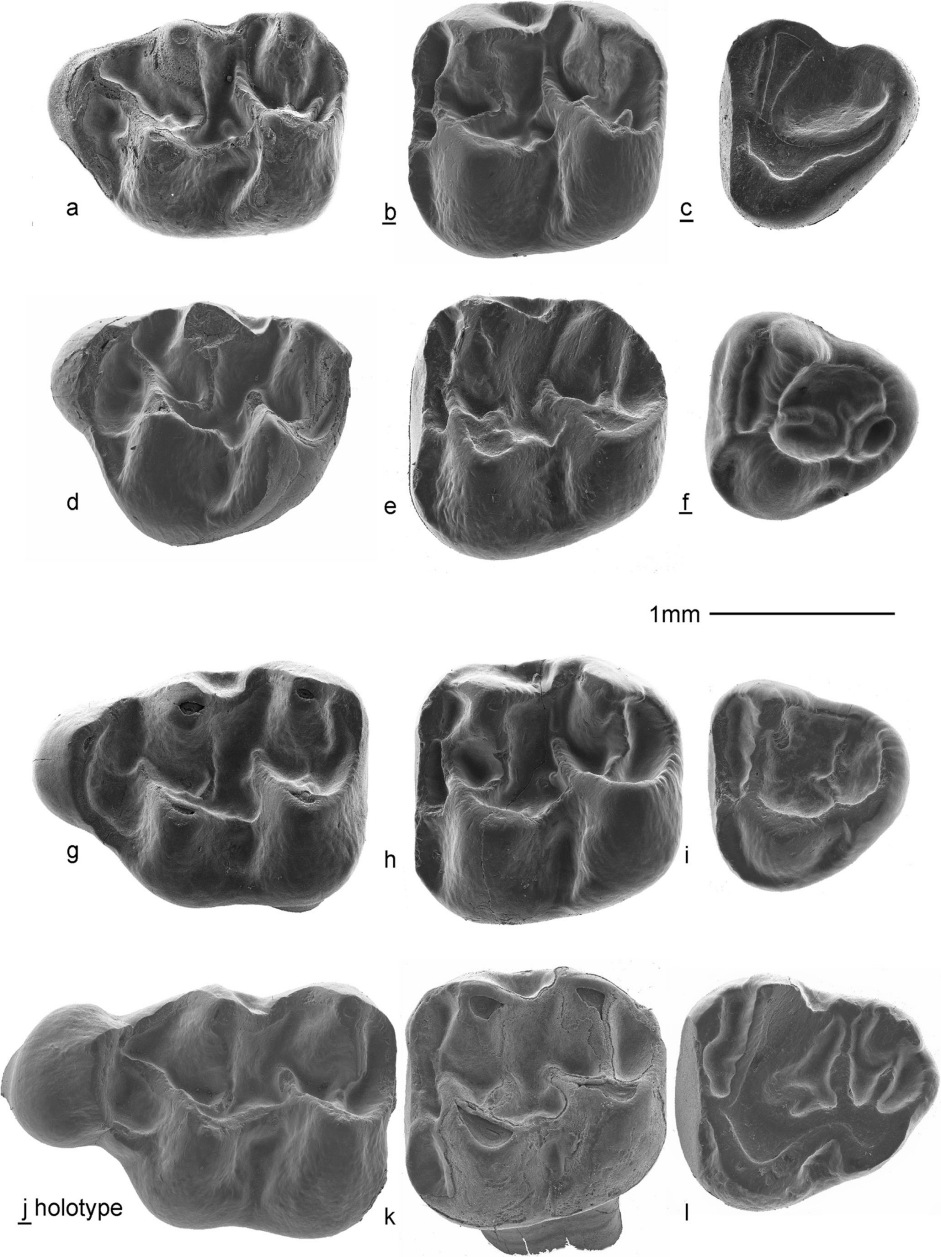
Upper dentition of *Paracricetodon stojanovici* nov. sp. and *P. gracilis* nov. sp*.* from Valniš. *Paracricetodon stojanovici* nov. sp.: **a** M1 sin VA-630, **b** M2 dext VA-667, **c** M3 dext VA-724, **d** M1 sin VA-652, **e** M2 VA-682, **f** M3 dext VA-723, **g** M1 sin VA-623, **h** M2 sin VA-678, **i** M3 sin VA-714. *Paracricetodon gracilis* nov. sp.: **j** (holotype) M1 dext VA-831, **k** M2 sin VA-834, **l** M3 sin VA-840

**Fig. 8:**
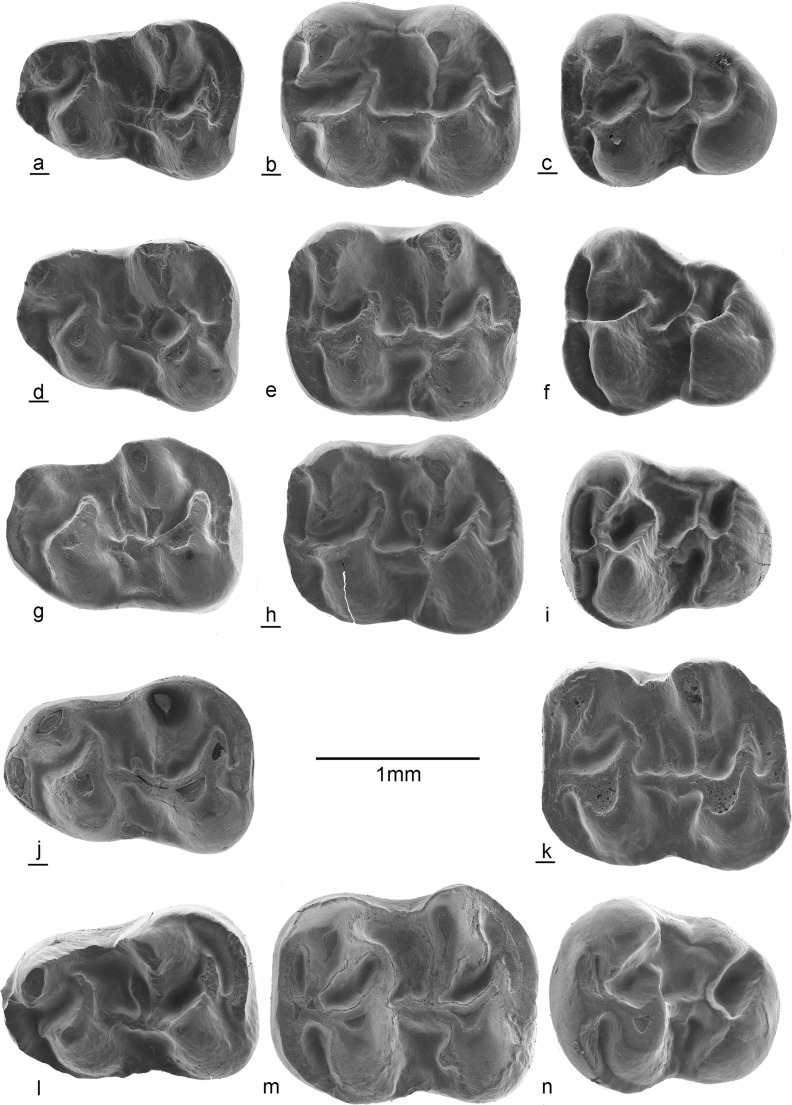
Lower dentition of *Paracricetodon stojanovici* nov. sp. and *P. gracilis* nov. sp. from Valniš. *Paracricetodon stojanovici* nov.sp.: **a** m1 dext VA-761, **b** m2 dext VA-782, **c** m3 dext VA-813, **d** m1 dext VA-742, **e** m2 sin VA-777, **f** m3 sin VA-1016, **g** m1 sin VA-740, **h** m2 dext VA-784, **i** m3 sin VA-821, **j** m1 dext VAL-020; *Paracricetodon gracilis* nov.sp.: **k** m2 dext VA-846, **l** m1 sin VA-841, **m** m2 sin VA-845, **n** m3 sin VA-848

**Material and measurements**: see Table 2 (Buštranje), Table 3 (Valniš), Table 4 (Strelac-1), Table 5 (Strelac-2) and Table 6 (Strelac-3). *Paracricetodon stojanovici* nov. sp. is represented in the Raljin collection by a fragment of a mandible with m1 – m3 and part of the lower incisor. These teeth could not be measured without destroying the specimen. Figures 5, 6, 7a–i, 8a–j and 10.

**Table 2:** Measurements of *Paracricetodon stojanovici* nov. sp. from Buštranje

	Length (mm)			Width (mm)		
**Buštranje**	**Range**	**Mean**	**N**	**Mean**	**Range**	**N**
M1	1.68–1.90	1.76	7	1.18	1.09–1.29	16
M2	1.25–1.47	1.36	20	1.23	1.17–1.30	19
M3	1.05–1.28	1.14	18	1.12	1.06–1.21	19
m1	1.40–1.52	1.46	13	1.01	0.94–1.07	17
m2	1.30–1.53	1.39	22	1.11	1.04–1.19	22
m3	1.27–1.48	1.38	19	1.07	0.99–1.15	19

**Table 3:** Measurements of *Paracricetodon stojanovici* nov. sp. from Valniš

	Length (mm)			Width (mm)		
**Valniš**	**Range**	**Mean**	**N**	**Mean**	**Range**	**N**
M1	1.72–2.01	1.86	20	1.27	1.11–1.37	33
M2	1.25–1.56	1.42	37	1.31	1.18–1.44	36
M3	0.93–1.25	1.09	38	1.12	0.99–1.21	37
m1	1.39–1.60	1.50	34	1.06	0.99–1.13	34
m2	1.41–1.65	1.53	24	1.20	1.10–1.28	26
m3	1.28–1.47	1.39	30	1.13	1.06–1.19	28

**Table 4:** Measurements of *Paracricetodon stojanovici* nov. sp. from Strelac-1

	Length (mm)			Width (mm)		
**Strelac-1**	**Range**	**Mean**	**N**	**Mean**	**Range**	**N**
M1	1.72–2.03	1.90	9	1.26	1.16–1.34	11
M2	1.33–1.52	1.44	15	1.33	1.22–1.43	14
M3	0.96–1.21	1.12	13	1.14	1.02–1.23	12
m1	1.42–1.58	1.50	8	1.07	0.94–1.14	8
m2	1.36–1.62	1.52	11	1.16	1.06–1.27	11
m3	1.30–1.62	1.44	8	1.18	1.00–1.26	8

**Table 5:** Measurements of *Paracricetodon stojanovici* nov. sp. from Strelac-2

	Length (mm)			Width (mm)		
**Strelac-2**	**Range**	**Mean**	**N**	**Mean**	**Range**	**N**
M1	1.72–2.08	1.90	6	1.31	1.18–1.39	7
M2	1.31–1.58	1.45	12	1.35	1.26–1.45	11
M3	1.06–1.18	1.10	7	1.14	1.09–1.14	7
m1	1.43–1.57	1.49	7	1.04	1.02–1.09	7
m2	1.40–1.51	1.46	5	1.16	1.09–1.27	5
m3	1.35–1.43	1.40	6	1.11	1.10–1.14	5

**Table 6:** Measurements of *Paracricetodon stojanovici* nov. sp. from Strelac-3

	Length (mm)			Width (mm)		
**Strelac-3**	**Range**	**Mean**	**N**	**Mean**	**Range**	**N**
M1	–	1.91	1	1.35	1.24–1.45	4
M2	1.34–1.51	1.42	7	1.31	1.20–1.42	7
M3	1.02–1.13	1.07	7	1.14	1.01–1.28	7
m1	1.40–1.65	1.49	9	1.05	0.98–1.18	9
m2	1.37–1.60	1.47	10	1.14	1.02–1.22	9
m3	1.32–1.35	1.34	3	1.11	1.07–1.17	4

**Diagnosis**: *Paracricetodon stojanovici* nov. sp. is small (Fig. 1). The M1, M2, M3, m1 and m2 are on average of the size of *Paracricetodon wentges*i but the M3 and m3 fall below or in the lower range of that species. The m3 is on average somewhat shorter than the m1 (Fig. 2).

The M1 has a crescent-shaped anterocone that is almost as high as the protocone and paracone. The M2 has a protolophule 1 and a, weaker, protolophule 2. The sinus of the sub-triangular M3 is either very small or absent. The anteroconid of the m1 is developed as a cingulum (anterolophid) that descends from the tip of the metaconid to the basis of the protoconid. The metalophulid 1 is absent and the metalophulid 2 is the posterior arm of the protoconid. The posterior arm of the hypoconid is more prominent than the metalophulid 2.

**Differential diagnosis**: *Paracricetodon stojanovici* falls in the size group of the small *Paracricetodon* species *P. wentgesi* and *P. gracilis*. All other species are considerably larger (Fig. 2). *Paracricetodon stojanovici* is very similar to *P. wentgesi* from Süngülü (late Eocene, Lesser Caucasus, Turkey). However, the M1, M2, m1 and m2 of *P. stojanovici* are on average somewhat smaller and the M3 and m3 are much smaller (Fig. 1) and have a more simple morphology than those of *P. wentgesi.* There are minor differences in the frequency distribution of the dental characters of the M1, M2, m1 and m2 between these species, but these are gradual and within the intraspecific variation of the two species. The main difference is that the most elaborate M3 of *P. stojanovici* has a simpler dental pattern than the most reduced M3 of *P. wentgesi.* A similar difference in the degree of reduction occurs in the m3 of these two species: the posterior part of this tooth is much narrower, the entoconid is lower and the mesolophid is absent in the specimens of *P. stojanovici.*

### Description of the material from the type locality Buštranje

**M1**: The shape of the occlusal surface as well as the dental pattern show considerable variation. The well-developed crescent-shaped anterocone has a labial position. The lingual and labial branch of the anterocone reached the bases of the protocone and paracone respectively in most specimens. The anterior arm of the protocone is strong and connected to the labial side of the anterocone in some specimens. The narrow, low protolophule 2 is incomplete in some M1. The posterior spur of the paracone is burgee-shaped and is not or weakly connected to the metacone. The weak longitudinal crest is formed by the posterior arm of the protocone and defines the shape of the posteriorly directed sinus. In some specimens this ridge does not quite reach the base of the hypocone. The strong anterior arm of the hypocone may reach the base of the paracone, but in the majority this ridge ends free. The more or less transverse metalophule shows a wide range in development: in most M1 it is weak, but in others it is quite strong. The posteroloph curves from the tip of the hypocone to the base of the metacone delimiting a long narrow posterosinus.

**M2**: The long, straight anteroloph is divided into a long labial branch and a shorter lingual branch by the very short anterolophule. The high protocone and paracone are connected by a strong protolophule 1 (= anterior arm of the protocone) and a weaker protolophule 2. These two lophs originate at the antero-labial corner of the protocone. The high hypocone and metacone are connected by a strong metalophule 2 and a weaker metalophule 1. These two lophs originate at the antero-labial corner of the hypocone.

The longitudinal ridge, which is formed by the posterior arm of the protocone, shows a considerable variation in height. The posterior spur of the paracone is burgee-shaped. The strong anterior arm of the hypocone reaches the base of the metacone in a few M2, in the others it ends freely in the main basin. The posteroloph connects the hypocone and metacone along the posterior limit of the occlusal surface.

**M3**: It shows a sub-triangular shape of the occlusal surface due to the near-absence of the hypocone. The anteroloph is divided into a long labial branch and a shorter lingual branch. The protocone, by far the largest cusp, is connected to the paracone by a straight, transverse protolophule. The pattern of the posterior part of the M3 shows considerable variation because of individual differences in the reduction of the hypocone, metacone and metalophule. In a few specimens, there is a remnant of the original longitudinal ridge preserved, but in the majority of the M3 the main basin shows some low cusps or ridges that are difficult to homologise. The hypocone and the metacone are incorporated into the posteroloph.

**m1**: It has a small anteroconid located on the anterolophid; it is much lower than the protoconid and the metaconid. In some m1 the anterolophid descends from the tip of the metaconid to the base of the protoconid. The high metaconid and entoconid are situated somewhat anteriorly of the protoconid and hypoconid. The metalophulid 1 is absent. The strong posterior arm of the protoconid reaches the base of the metaconid (forming a metalophulid 2) in most m1, in the others this ridge ends free in the main basin. The metaconid and the entoconid are connected by a narrow ectolophid. The longitudinal ridge is low, but complete and may bear a weak mesolophid and ectomesolophid. The low hypolophulid is directed slightly forwards and inserts on the longitudinal ridge. The strong posterior arm of the hypoconid reaches the base of the entoconid in some specimens, but in others it ends free. The posterolophid is complete and lower than the posterior arm of the hypoconid.

**m2**: The curved labial branch of the anterolophid is shorter than the straight lingual branch. The high metaconid and entoconid are situated somewhat anteriorly of the protoconid and hypoconid. The short, weak metalophulid is almost transverse and inserts on the anterolophule in front of the protoconid. The protoconid and hypoconid are connected by the longitudinal crest and the metaconid and entoconid are connected by an ectolophid. The strong posterior arm of the protoconid reaches the metaconid in some specimens (forming a metalophulid 2), but in others it ends free. A weak mesolophid is present in some specimens. The hypolophulid is more or less transverse and inserts on the longitudinal ridge in front of the hypoconid or is connected to the anterior part of the hypoconid. The posterior arm of the protoconid and the hypoconid have about the same length, except in some m2 where the posterior arm of the hypoconid reaches the base of the entoconid, forming a hypolophulid 2. The posterolophid curves smoothly from the posterior side of the hypoconid to the entoconid.

**m3**: The short anterolophulid divides the anterolophid into a short curved labial branch and a longer straight lingual branch. The metaconid and entoconid are situated slightly anteriorly of the protoconid and hypoconid. The metalophulid 1 is approximately transverse and inserts on the anterolophulid or on the anterior part of the protoconid. The strong posterior arm of the protoconid ends freely in the majority of the m3, but reaches the base of the metaconid in some others. The mesolophid is absent. The short, transverse hypolophulid inserts on the longitudinal ridge in front of the hypoconid. The metaconid is connected to the weak entoconid by an ectolophid. The posterior arm of the hypoconid is absent. The posterolophid is connected to the entoconid.

### Remarks

The *Paracricetodon stojanovici* nov. sp. molars from Valniš, Strelac-1, Strelac-2, Strelac-3 and Raljin do not differ in size; the *Paracricetodon stojanovici* nov. sp. molars from the type locality Buštranje (Eocene) tend to be slightly smaller than those of the Oligocene localities in the Strelac area (Fig. 6) except for the M3. The M3 of the Oligocene populations (black symbols in Fig. 6) have more smaller specimens suggesting a trend toward reduction in size. Morphologically the populations from Valniš, Strelac-1, -2, -3 and Raljin are within the range of variation of the collection from the type locality. The similarity in morphology and the somewhat larger size of the younger samples suggest an ancestor, descendant relationship, but the slight size difference does not warrant specific separation. The material of *Paracricetodon stojanovici* nov. sp. differs from *P. wentgesi* mainly by its smaller size (Figs. 1, 2 and 3) and the simpler dental pattern of the M3/m3. Otherwise the difference between these species is limited to differences in frequency of such morphological character states as the length of the anterior arm of the protocone in the M2 and the frequency of a forwards directed metalophulid 1 in the m2. The similarity of *P. stojanovici* nov. sp. and *P. wentgesi* suggests that these species are closely related, but there does not seem to be a direct ancestor – descendant relationship, because, while we see size increase through time in the Serbian material, *P. stojanovici* nov. sp. from the Oligocene is smaller and has more reduced M3/m3 than *P. wentgesi* from the late Eocene of Turkey. Although our age control is poor there are good reasons to assume that the locality Süngülü is about coeval with Buštranje and older than the localities in the Strelac area.

*Paracricetodon gracilis* nov. sp.

(Fig. 7j–l; Fig. 8k–n)

**Derivatio nominis**: gracilis in Latin means slender. The M1 of this species is relatively long and narrow for a paracricetodontine.

**Type locality**: Valniš.

**Holotype**: One isolated M1dext. VA-831 (Fig. 7j).

**Occurrences**: Strelac-3.

**Material and measurements:** Valniš: Table 7 and Figs. 1, 6, 7j–l and 8k–n, Strelac-3: Two m2; L x W: 1.68–1.77 mm; 1.30–1.40 mm.

**Table 7:** Measurements of *Paracricetodon gracilis* nov. sp. from Valniš

	Length (mm)			Width (mm)		
**Valniš**	**Range**	**Mean**	**N**	**Mean**	**Range**	**N**
M1	2.20–2.21	2.21	2	1.38	1.37–1.38	2
M2	1.52–1.60	1.56	2	1.40	1.37–1.43	2
M3	–	1.49	1	1.32	–	1
m1	1.62–1.63	1.63	2	1.19	1.12–1.22	3
m2	1.62–1.72	1.67	3	1.32	1.28–1.35	2
m3	1.46–1.59	1.54	3	1.19	1.15–1.21	3

**Diagnosis**: The M1, M2, M3, m1 and m2 are on average intermediate in size between those of the larger *Paracricetodon walgeri* and the smaller *P. wentges*i and *P. stojanovici*. The length of the M2 overlaps the range of *P. wentgesi* and the length ranges of the m1, m2 and m3 overlap with those of *P. stojanovic*i and *P. wentgesi*. The narrow anterocone of the M1 is situated far forwards, which gives this tooth a, for *Paracricetodon* unusual, gracile appearance. The dental pattern of the M3 is not much reduced and shows a hypocone, metalophule and mesoloph. The m3 is on average shorter than the m1.

**Differential diagnosis**: *Paracricetodon gracilis* differs from all other *Paracricetodon* species by having an elongate gracile M1 and from all other species except *P. stojanovici* by having an m3 that is shorter than the m2. Although the morphology of the cheek teeth of *P. gracilis* is essentially similar to that of other species of *Paracricetodon,* the length/width ratio of its M1 in combination with a relatively short m3 are unique.

### Description of the type material from Valniš

**M1**: The rather narrow anterocone is situated at about the same distance from the protocone and paracone as there is between the latter cusps and the metacone and hypocone. The four main cusps have about the same size and height. The anterocone, paracone and metacone are connected along the labial margin by an ectoloph. The anterior arm of the protocone reaches the postero-labial base of the anterocone. The somewhat shorter anterior arm of the hypocone does not reach the ectoloph. The lingual branch of the anterocone connects this cusp to the protocone. The shape of the sinus is determined by the posterior arm of the protocone. The short, weak protolophule and metalophule are slightly forwards directed and insert on the protocone and hypocone, respectively. The posteroloph descends from the hypocone and ascends labially to the metacone.

**M2**: The occlusal surface is sub-square. The low anteroloph occupies all of the anterior border of the M2 and continues as a cingulum to the postero-lingual side of the protocone and to the postero-labial side of the paracone. The four main cusps are large and the lophs are subordinate to the cusps. The parallel anterior arms of the protocone and hypocone are strong and end free. The short protolophule and metalophule are directed forwards and insert on the protocone and hypocone, respectively. The posterior spur of the paracone is strong and forms an ectoloph. The sinus is delimited by the posterior arm of the protocone. The posteroloph descends from the hypocone and ascends labially to the tip of the metacone.

**M3**: The dental pattern of the elongate M3 is not reduced. The narrow anteroloph is straight. The protocone is larger than the prominent hypocone. The protocone and hypocone are connected by the posterior arm of the protocone. The paracone is larger than the prominent metacone. The forwards directed protolophule and metalophule are both complete. The anterior arm of the hypocone forms a long straight mesoloph, which reaches the labial border of the occlusal surface.

**m1**: The anteroconid is developed as a ridge that is much lower than the protoconid and metaconid. This incipient anteroconid hardly adds to the length of the m1. The four main cusps are robust, but the longitudinal crest and ectolophid are weak. The posterior arm of the protoconid is connected to the base of the metaconid, but the posterior arm of the hypoconid ends free. The short transverse hypolophulid is weak. The posterolophid descends from the hypoconid and ascends lingually to the tip of the entoconid. The mesolophid is weak.

**m2**: The labial branch of the anterolophid is longer than the lingual branch. The metalophulid 1 and the hypolophulid are directed somewhat anteriorly and insert in front of the protoconid and hypoconid, respectively. The parallel posterior arms of the protoconid and hypoconid are about equally strong, but the former connects to the base of the metaconid, while the latter ends free. The metaconid and entoconid are connected by an ectolophid and the protoconid and hypoconid are connected by the longitudinal crest, which delimits the more or less symmetrical sinusid. The posterolopid descends from the hypoconid and ascends lingually to the tip of the entoconid.

**m3**: Recognition of the isolated m3 of *Paracricetodon gracilis* nov. sp. poses a problem, because that supposedly small tooth is only very slightly larger than the m3 of *P. stojanovici.* One of the three m3 (VA-848, Fig. 8n) that we tentatively allocate to *P. gracilis* is somewhat more robust than the other two and is therefore with confidence considered to belong to *P. gracilis*. Our description of the m3 is therefore based on this specimen. The labial branch of the anterolophid is much stronger than the lingual branch and continues as a well-delimited cingulum along the labial side of the protoconid. The cusps are robust and the short, low metalophulid and hypolophulid are directed forwards. The posterior arm of the protoconid ends free in the main basin. The posterior arm of the hypoconid is absent. The entolophid and ectolophid are low, but the strong posterolophid is connected to the well-delimited entoconid.

### Remarks

The type material of this species is unfortunately somewhat meagre, due to limited access to the locality. Reason for the lack of an adequate collection of *P. gracilis* is that we had to pay a considerable amount of money to the land owner of the Valniš site in order to obtain his permission for further collecting, which we refused. As a result there remains some uncertainty about the allocation of the lower cheek teeth to *Paracricetodon gracilis.* Our decision to regard *P. gracilis* as a separate species is based on the size as well as on the morphology of the upper cheek teeth. Moreover, the sole complete M1, the three M2 and the unique M3 are neatly intermediate in size between the teeth of the larger *P. dehmi* and *P. walgeri* and the smaller *P. wentgesi* and *P. stojanovici* (Fig. 1). The identification of the lower cheek teeth of *P. gracilis* is more problematic, because their size difference relative to *P. gracilis* and *P. stojanovici* is small. It can therefore not be excluded that the identification of the lower cheek teeth of *P. gracilis* is not correct, but concerns large specimens of *P. stojanovici*.

In our experience, the relative size of the cheek teeth within rodent genera is rather stable. This even so when details of the dental pattern change through time. If our allocation of the lower cheek teeth to *P. stojanovici* and *P. gracilis* is correct these two species from Serbia have m3 that are shorter than the m2, while all other species of *Paracricetodon* have m3 that are longer than the m2 (Fig. 3).

**Fig. 9:**
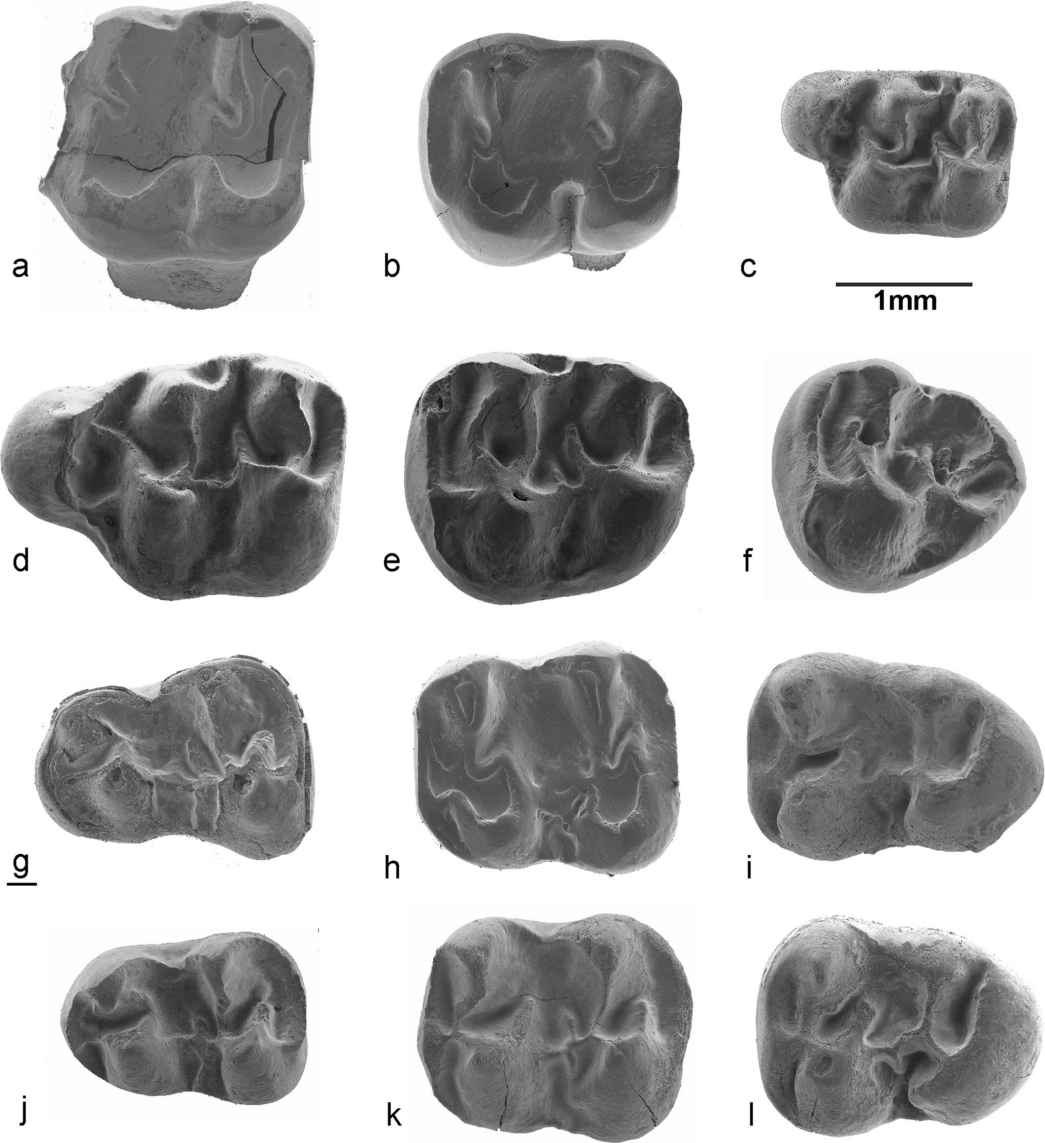
*Paracricetodon dehmi* from Valniš, Strelac-3 (Serbia) and Belgarite (France). For comparison of size, a M1 of *P. stojanovici* nov. sp. (**c** BUS-682) has been added to the figure. *Paracricetodon dehmi* from Valniš and Strelac-3: **a** M1 sin STR-1-281, **b** M2 sin VA-856, **g** m1 dext VA-862, **h** m2 sin VA-866, **i** m3 sin STR1-288. *Paracricetodon dehmi* from Belgarite (Fr): **d** M1 sin BEL-401, **e** M2sin BEL-403, **f** M3 sin BEL-404, **j** m1 sin BEL-403, **k** m2 sin BEL-406, **l** m3 sin BEL-407

*Paracricetodon dehmi* Hrubesch, 1957(Fig. 9a, b, g, h, i)

*P. kavakderensis* Ünay-Bayraktar, 1989

*P. kodjayarmensis* Ünay-Bayraktar, 1989

*P.* cf. *kavakderensis* (in Doukas and Theocharopoulos 1999)

**Type locality**: Bernloch (Germany).

**Occurrences**: Kavakdere and Kocayarma (Turkish Thrace), Kyprinos (Greek Thrace), Belgarite 4A (Quercy, France), Valniš and Strelac-1 (south-east Serbia).

**Material and measurements**: Valniš: Table 8 and Figs. 1, 9a, b, g–i, Strelac-1: Table 9.

**Table 8:** Measurements of *Paracricetodon dehmi* from Valniš

	Length (mm)			Width (mm)		
**Valniš**	**Range**	**Mean**	**N**	**Mean**	**Range**	**N**
M2	1.87–2.18	2.01	3	1.84	1.71–1.95	3
M3	–	1.77	1	1.69	–	1
m1	1.94–2.15	2.03	3	1.45	1.40–1.53	3
m2	2.04–2.08	2.06	2	1.60	1.56–1.64	2

**Table 9:** Measurements of *Paracricetodon dehmi* from Strelac-1

	Length (mm)			Width (mm)		
**STR-1**	**Range**	**Mean**	**N**	**Mean**	**Range**	**N**
M1	–	–	–	1.88	–	1
m1	–	2.03	1	1.45	–	1
m2	–	2.11	1	1.66	–	1
m3	2.20–2.28	2.24	2	1.63	1.55–1.70	2

### Description of the specimens

**M1**: The only M1 from south-east Serbia (Fig. 9a) is damaged anteriorly. The short anterior arms of the protocone and hypocone are parallel. The transverse protolophule and metalophule insert on the protocone and the hypocone, respectively. The sinus is directed posteriorly. The posterior spur of the paracone reaches the base of the metacone. The posteroloph is connected to the metacone.

**M2**: The occlusal surface of the three M2 from Valniš is somewhat narrower posteriorly than anteriorly. The weak anteroloph of two M2 is not divided by an anterolophule into a lingual and labial branch. The parallel, forwards directed anterior arms of the protocone and hypocone are rather short and end free. The short protolophule and metalophule bend slightly forwards and insert on the protocone and hypocone, respectively. The posteroloph is connected to the metacone.

**M3**: The only M3 available is unfortunately rather worn. The anteroloph of this sub-triangular tooth is weak. The protocone has a short free ending anterior arm. The protolophule connects the paracone to the protocone and there are remnants of the metalophule. The hypocone is very small and the sinus is shallow.

**m1**: The “anteroconid” is a low cingulum that hardly adds to the length of the m1 (Fig. 9g). An anterolophulid divides the weak anterolophid into a short lingual and a somewhat longer labial branch. A true metalophulid 1 is missing and the short metalophulid 2 is formed by the posterior arm of the protoconid. The longitudinal crest is low. Two of the three specimens show a small ectomesolophid as well as a double mesolophid, but these structures are absent in the third. The low ectolophid is formed by the posterior spur of the metaconid and the anterior spur of the entoconid. The posterior arm of the hypoconid is short and ends free. The posterolophid descends from the hypoconid and ascends lingually to the tip of the entoconid.

**m2**: Two of the three m2 are so badly worn that the details of the dental pattern are not visible. The third one (Fig. 9h) has robust cusps, but a weak anterolophid. The ectolophid and longitudinal crest are low. The metalophulid and hypolophulid are transverse and insert on the anterior side of the protoconid and hypoconid, respectively. The parallel posterior arms of the protoconid and hypoconid are short and end free. There is a trace of a mesolophid. The rather low posterolophid reaches to the tip of the entoconid.

**m3**: The two m3 in the size category of *Paracricetodon dehmi* are rather different. Number STR-1-288 (Fig. 9i) is elongate and narrow posteriorly like the ones from Bernloch (Hrubesch, 1957). The other (STR-1-289, not illustrated) is much wider posteriorly, lacks the strong posterior arm of the protoconid and shows traces of irregular ridges within the central basin. This specimen resembles *Trakymys saratji* Ünay-Bayraktar, 1989 much more than *Paracricetodon dehmi.* Our tentative allocation of this m3 to the latter species is induced by our reluctance to enter a taxon to the fauna list which is based on one, possibly aberrant, m3 only. The description is based on the slender specimen. The anterolophid is low and divided by the anterolophulid in a short labial and a longer lingual branch. The metalophulid and the hypolophulid are directed slightly forwards and insert in front of the protoconid and hypoconid, respectively. The posterior arm of the protoconid ends free. The low ectolophid bears a distinct mesoconid. The posterior arm of the hypoconid is absent. The posterolophid connects the hypoconid to the entoconid.

### Remarks

The teeth from Valniš and Strelac-1 allocated to *Paracricetodon dehmi* match the ones in the type series from Bernloch in every respect. A reconstructed dentition of *P. dehmi* from Belgarite 4A is figured for comparison (Fig. 9d–f, j–l), because the photographs of the type material given by Hrubesch (1957), antedating the invention of the scanning electron microscope, are not very clear.

## The lower incisors

**Fig. 10:**
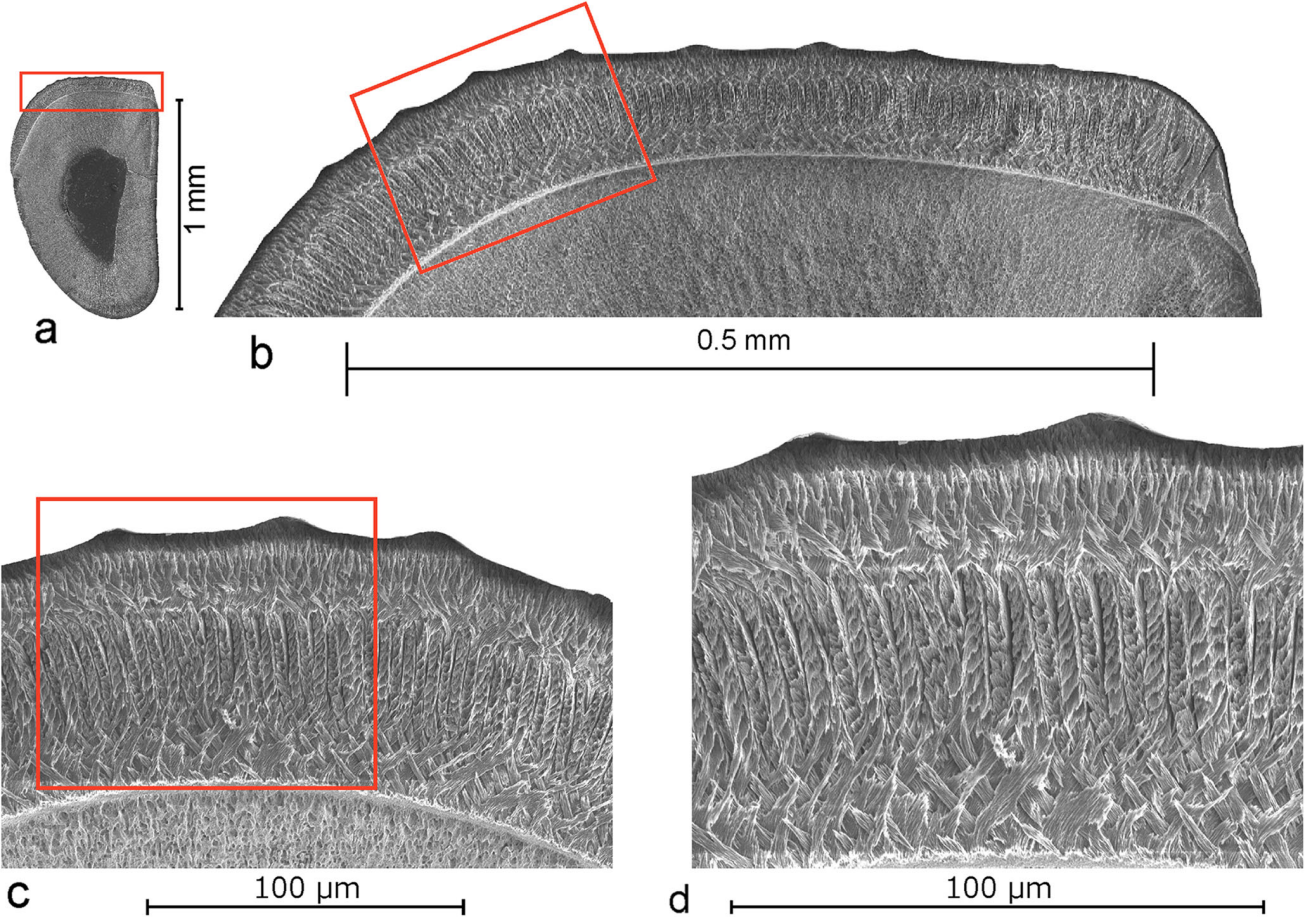
The lower incisor of *Paracricetodon stojanovici* nov. sp. from Buštranje. Shown are **a** transverse section, **b** detail showing the external surface with ridges, **c** detail of **b**, **d** detail of **c**

Matching isolated incisors with the cheek teeth is often difficult and a possible source of mistakes. This in particular so when the associations studied contain a diverse array of Muridae subfamilies as in the localities of the Strelac area and in Buštranje. Fortunately the Paracricetodontinae studied so far by Kalthoff (2000, 2006) are known to have a very derived four-layered schmelzmuster in the lower incisor and are therefore potentially readily recognisable. Testing transverse sections of the various incisor types from Buštranje showed that the, for *Paracricetodon* characteristic specialised type 8 enamel (Kalthoff 2000), occurs in lower incisors from Buštranje. The outer enamel surface of these specimens shows seven to eight longitudinal, distally somewhat tangential ribs (Fig. 10b). Similar ribs are illustrated by Hugueney and Adrover (1989-1990). The HSB are longitudinally oriented. The transverse section shows that the basal part of the PI consists of a thin layer of enamel with the IPM at an angle to the prisms and a much thicker upper part with prism parallel IPM. The relatively thin PE consists of an internal layer of tangential enamel and an external layer of radial enamel (Fig. 10c, d).

The occurrence of this very derived schmelzmuster in the presumably late Eocene locality Buštranje shows that the major radiation that led to the diversity of the Oligocene Muridae dates at least back to the middle Eocene.

## Biostratigraphy

The paracricetodontines do not provide much information on the age of the Serbian localities, because two of the three species described above are not known from elsewhere and the stratigraphical range of *Paracricetodon dehmi*, originally from Bernloch, a locality correlated with MP 25, is not known. *P. stojanovici* is very similar to *P. wentgesi* from Süngülü, a locality which is supposed to have an age around the Eocene-Oligocene boundary. However, our species, which has presumably a similar age has a more reduced M3/m3 and is slightly smaller. Differences that may be due to adaptations to different ecological niches rather than due to difference in age.

## Conclusions

*Paracricetodon* is present with about 10% in the late Eocene rodent fauna of Buštranje, is common (30–70%) in the early Oligocene rodent faunas of Strelac, Raljin and Valniš. The rodent assemblages from Valniš and Strelac-3 contain three species of *Paracricetodon.* The assemblage from Strelac-1 shares two of these and further contains one m3 that possibly represents the genus *Trakymys.* Such a diversity of species and abundance of specimens of paracricetodontines is not known from elsewhere and suggests that the group underwent radiation on the Serbo-Macedonian land area. The new species *Paracricetodon stojanovici* is described from Buštranje and *P. gracilis* from Valniš. *Paracricetodon stojanovici*, a common constituent in its type locality, is also present in the collections from the younger localities Valniš, Strelac-1, -2, -3 and Raljin, while the rare *P. dehmi* occurs in Valniš and Strelac-1 and *P. gracilis* has been found in Valniš and Strelac-3 only.

A review of *Paracricetodon* species suggests that the hitherto formally described species *P. spectabilis*, *P. cadurcensis*, *P. dehmi*, *P. walgeri* and *P. wentgesi* are primarily distinct in size. If the upper dentition of the inadequately known *P. spectabilis* figured in Schaub (1925) and Stehlin and Schaub (1951) will appear to be a modal specimen, this species is not only the largest, but also the only *Paracricetodon* species with an M3 that is almost the same length as the M2. *Paracricetodon kodjayarmensis* and *P. kavakderensis* are considered to be junior synonyms of *P. dehmi* and *P. confluens* is considered a “Nomen Dubium”.

The late Eocene newly described *Paracricetodon stojanovici* has very derived email pattern in its lower incisor showing that the major radiation that led to the generic diversity of the Oligocene Muridae dates at least back to the middle Eocene.
